# Efficacy of orally and topically administered fluralaner (Bravecto^®^) for treatment of client-owned dogs with sarcoptic mange under field conditions

**DOI:** 10.1186/s13071-020-04395-6

**Published:** 2020-10-17

**Authors:** Rafael Chiummo, Ivo Petersen, Claudia Plehn, Eva Zschiesche, Rainer Roepke, Emmanuel Thomas

**Affiliations:** MSD Animal Health Innovation GmbH, Schwabenheim, Germany

**Keywords:** Bravecto^®^, dog, field, fluralaner, sarcoptic mange, *Sarcoptes*

## Abstract

**Background:**

Successful canine sarcoptic mange treatment requires immediate efficacy to eliminate active mites, and sustained activity to prevent re-infestation from in-contact animals and fomites. With extended acaricidal activity, fluralaner has been shown to be effective for treating this disease. To confirm this potential under field conditions, two fluralaner formulations were administered to mite-infested, client-owned dogs.

**Methods:**

Households qualified for inclusion if they had at least one dog positive for *Sarcoptes scabiei* mites, confirmed by skin scraping, and at least one dog with clinical signs evocative of sarcoptic mange. Households were allocated to groups of dogs to receive a single treatment with either oral (Bravecto^®^ chewable tablets, MSD Animal Health) or topical (Bravecto^®^ Spot-on, MSD Animal Health), fluralaner at a dose of ≥ 25 mg/kg (range 25–56 mg/kg) on Day 0, or two treatments with oral sarolaner (Simparica^®^ tablets, Zoetis) (Days 0 and 28) at ≥ 2 mg/kg (2–4 mg/kg). All dogs in each household were treated with the same product. On the enrolment day and subsequently on Days 28, 56 and 84, deep skin scrapings were taken from at least five different body areas judged to be most likely to have active mite infestation. At each visit, the dog’s mange-associated skin lesions were recorded, and pruritus level was assessed.

**Results:**

There were 98 participating households and 135 dogs enrolled across Albania, France, Italy and Portugal. On Day 28, more than 90% of dogs in each group were negative for mites. On Days 56 and 84, all study dogs were free of mites and most dermatological signs of sarcoptic mange had resolved. There were no treatment-related adverse events in any group.

**Conclusions:**

A single treatment of client-owned, sarcoptic mange-affected dogs with either fluralaner chewable tablets or fluralaner spot-on formulation proved a safe and effective treatment of infestations with *S. scabiei* var. *canis*, maintained through 84 days (12 weeks) after treatment.
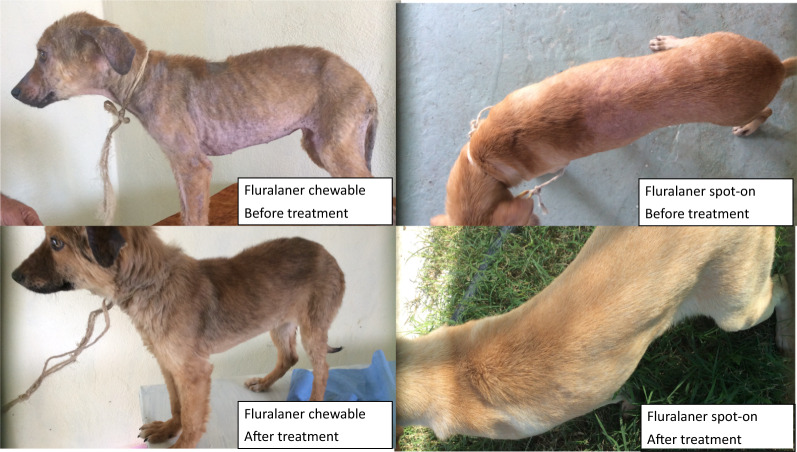

## Background

Canine scabies (sarcoptic mange) is a non-seasonal, intensely pruritic, transmissible infestation of the skin of dogs caused by the mite *Sarcoptes scabiei* var. *canis* [1]. Clinical presentation is characterized by intense pruritus and scratching, alopecia, inflammation, excoriation and hyperkeratosis, potentially associated with secondary bacterial infection and pyoderma. The disease occurs globally and can affect dogs of all breeds, age and either sex. The infestation is highly contagious; and both dogs and their human companions can develop skin lesions after contacting an infested host [1–3]. Infestation can also occur from contact with a fomite as mites can survive off a canine host for up to 21 days, depending on ambient temperature and relative humidity, with cooler temperatures and lower humidity prolonging survival [1, 4]. Scabies mites cause disease in other species as well, and the World Health Organization (WHO) includes scabies, caused by *S. scabiei* var. *hominis* on its list of human neglected tropical diseases: [1, 4–6]. Female mites that have found a host mate on the skin surface and then penetrate the stratum corneum, forming tunnels in which they lay eggs that hatch 2 to 3 days later. Completion of the mite life-cycle, egg to adult, has been estimated at 15 to 21 days [1, 4].

Effective control therefore depends on use of products that kill all motile parasite stages on the host, with sufficient duration of activity to ensure that eggs hatching subsequent to treatment do not produce viable larvae. To prevent re-infestation from the dog’s environment, that activity duration should also persist beyond the *S. scabiei* off-host survival time. Acaricides that act by contact following topical application have limited efficacy against *S. scabiei* because of the difficulty in penetrating deep into the female mite’s tunnels in the stratum corneum, and because their efficacy duration can be reduced by the use of adjunctive shampoo therapy and bathing to remove crusts and dermal debris [1]. Systemically acting acaricides, such as orally or topically administered fluralaner, are not affected by these considerations [7–9].

Therefore, an effective miticide should provide sufficient duration of activity to ensure that any eggs hatching subsequent to treatment do not produce viable larvae, and to prevent re-infestation from the dog’s environment. Additionally, all in-contact hosts must be effectively treated to prevent subsequent re-infestation. Although variants of *S. scabiei* are largely species-specific, *S. scabiei* var. *canis* can survive and occasionally establish in non-canine hosts, so in addition to treating in-contact dogs, all in-contact non-canine mammals should be evaluated as potential sources of re-infestation [1]. For these reasons, a systemically acting miticide with a sustained activity is important in the treatment and control of sarcoptic mange.

Fluralaner is a long-acting isoxazoline, approved in different formulations in more than 80 countries for the treatment and control of flea and tick infestations of dogs and cats, and for the treatment of poultry mite infestations. In many countries, fluralaner is also approved for the treatment of canine generalized demodicosis and feline ear mite infestations [10]. Following administration to dogs at 25 mg/kg of body weight, fluralaner has a half-life of 12 to 14 days (oral) and 17 to 21 days (topical) [11, 12]. The potent and persistent acaricidal and pharmacokinetic properties of fluralaner suggest this compound could be effective for treatment of sarcoptic mange in dogs. This was demonstrated in a South African study in which naturally infested, client-owned dogs were administered fluralaner either orally (n = 9) or topically (n = 11) [13]. All mites were eliminated from treated dogs within 4 weeks following a single treatment, while all untreated controls (n = 9) remained infested. In an uncontrolled study in Mexico, scrapings from 17 dogs diagnosed with sarcoptic mange were all negative for mites from 14 days after receiving orally administered fluralaner through the final observation on Day 28. Improvements in pruritus and lesion severity were observed within 14 to 21 days post-treatment [14]. To confirm the efficacy of fluralaner, an extended duration study was undertaken under European field conditions, involving client-owned dogs diagnosed with sarcoptic mange. Observations in this study extended to 84 days (12 weeks) following fluralaner administration, which is the expected duration of acaricidal effect after a single administration, based on previous studies [10–12].

## Methods

This multi-center, positive-controlled field study was conducted in alignment with the Good Clinical Practice, VICH Guideline 9 [15], Guideline on Statistical Principles for Veterinary Clinical Trials (EMA/CVMP/EWP/81976/2010) [16] and with the Guideline for the Demonstration of Efficacy of Ectoparasiticides (7AE17a, Sep 1994) [17] at 9 veterinary clinics across Albania, France, Italy and Portugal. At each site, the study activities were distributed between an unmasked Dispenser, who was responsible for treatment assignments and administrations, but took no part in clinical assessments, and an Examiner who was masked and made all treatment-related observations, including skin scrapings and skin lesion evaluation.

### Enrolment of dogs

Dogs with clinical signs of sarcoptic mange (pruritus, papules, crusts, erythema) and at least one live *S. scabiei* mite (larva, nymph, adult) found in at least one deep skin scraping qualified for the study [13]. Any dogs sharing a household with an enrolled dog were treated with the same product, regardless of the presence or absence of a positive skin scraping or clinical signs. Every household dog with at least one live *S. scabiei* mite before treatment was evaluated for efficacy, but mite-negative dogs were not monitored for infestation status and were included in the safety evaluation only (additional dogs).

Dogs remained at home during the study. Grooming and bathing were not permitted within 3 days after the initial treatment (Day 0) but were allowed for all groups on all subsequent study days. Dogs were not included if they received any medication, such as macrocyclic lactones, that might interfere with the efficacy assessment. During the study, dog treatment with any products with insecticidal/acaricidal or insect growth regulator activity was not permitted. No premises sanitation with products having acaricidal or insecticidal activity was allowed within 2 months before study start or during the study.

### Treatments

Each dog was allocated randomly to one of three treatment groups, in a ratio of 2:2:1, to receive oral fluralaner (Bravecto^®^ chewable tablets, MSD Animal Health, Schwabenheim, Germany), topical fluralaner (Bravecto^®^ Spot-on, MSD Animal Health), or oral sarolaner (Simparica^®^, Zoetis, Louvain-la-Neuve, Belgium), respectively. All treatments were administered in accordance with label directions, and all dogs within a study household were treated on the same day. Fluralaner was administered once at the approved dose rate of 25–56 mg/kg on Day 0, while sarolaner was administered at 2–4 mg/kg on Days 0 and 28 (4 weeks later) according to its approved label in Europe against *S. scabiei*. Dogs showing clinical signs of bacterial pyoderma received oral antibiotic treatment where recommended by the Examiner (two dogs, one in the sarolaner group, and one in the fluralaner spot-on group, required this treatment). Owners were instructed to carefully observe their dog(s) and advise the clinic of any unfavorable events between each scheduled visit.

### Assessments of sarcoptic mange

At the enrolment visit (Day 0) and at subsequent visits on Days 28, 56 and 84, deep skin scrapings were taken from at least five different body areas that showed the most severe lesions (crusts, alopecia, erythema, papules) suggesting the likely presence of local mite infestation. Each scraping, taken over an area of approximately 2.5 cm^2^, was made in the direction of hair growth with a blade or spatula coated with mineral oil, to an approximately constant depth so that capillary oozing was evident. When obtaining a scraping was difficult (area difficult to access), hairs were plucked from an affected area and placed in mineral oil. The scraping was examined for the presence of live mites under a stereomicroscope and the assessment of the scraping results was based on mite counts and clinical signs. If no mites were detected in five scrapings in a dog showing clinical signs, then additional scrapings were made until live mites were found, or until the maximum of 10 scrapings was reached. If no mites were detected in the first five scrapings in dogs without clinical signs, then no additional scrapings were made. The total number of mites counted in all scrapings was recorded. If needed, dogs could be sedated to allow effective skin scraping. Subsequent to Day 0, the same affected area was scraped on each visit if possible. However, if the area of the previous scraping appeared normal and other lesions were present, then the new lesions were scraped. When there were no observed lesions, the previous lesional areas were scraped.

At each scheduled visit, and before skin scraping (and treatment), the presence and severity of skin lesions, i.e. erythema, pustules/papules, crusts/scales, alopecia and any other dermatological signs, were recorded. The Examiner also scored each dog’s level of pruritus using a scale of 0 (no pruritus) to 10 (extreme pruritus).

### Assessing results

Primary efficacy was determined from the percentage of dogs free of live mites at the last evaluation (Day 84, week 12) for each study group [17]. Secondary efficacy parameters were determined from the percentage of dogs free of live mites on Days 28 and 56, and the regression of pruritus, alopecia and other skin lesions was evaluated.

## Results

A total of 135 dogs from 98 households were included across Albania (*n* = 50), France (*n* = 43), Italy (*n* = 15) and Portugal (*n* = 27). An additional 19 in-contact but mite-negative dogs in study households received study product and were evaluated only for safety. In these households, all dogs received the same product. The majority of dogs were mixed breed (60%), with 40% of dogs recorded as pure-bred. Most study dogs in each group were from households with a single dog (70%), with two households in the oral fluralaner group and one household in each of the topical fluralaner and oral sarolaner groups having five dogs. There were no treatment-related adverse events reported in any group.

Protocol violations resulted in the exclusion of 9 dogs, leaving 126 dogs for efficacy assessments. Study dogs ranged in age from 10 weeks to 11 years (median 3.0), weighed from 2.8 to 52.0 kg (median 12.8), and included 61 females (70.5% spayed) and 61 males (64.6% neutered). The protocol violations applied to three dogs in the topical fluralaner group that were not presented according to visit schedules, one dog in the oral fluralaner group that was administered ivermectin during the study, and five dogs in the oral sarolaner group, all from a single household, that died between Days 0 and 28 as a result of massive infections with *Toxocara canis*, confirmed by necropsies.

On Day 0, mean mite counts were 9.6, 10.4, and 10.3 in the oral fluralaner, topical fluralaner and oral sarolaner groups, respectively, with maximum counts of 55, 86 and 35 mites (Table 1). One dog from the oral fluralaner group did not present with alopecia, one dog from the topical fluralaner group did not present with pruritus, and one dog from the oral sarolaner group did not present with lesions. The most commonly observed clinical signs of sarcoptic mange in all groups at enrolment were pruritus (99%), alopecia (99%) and crusts or scales (100%). Erythema (78%), and pustules or papules (76%) were also commonly observed before treatment. Additionally, 2 dogs presented wounds, another 2 ulceration and one dog had excoriation.

**Table 1:** Initial homogeneity of sarcoptic mange parameters in study dogs

	Oral fluralaner (*n* = 54)	Topical fluralaner (*n* = 46)	Oral sarolaner (*n* = 26)
Initial mite counts			
Mean ± SD	9.6 ± 10.5	10.4 ± 14.3	10.3 ± 10.2
Median	5.5	5.5	6.0
Range	1–55	1–86	1–35
Initial pruritus scores	6.0 ± 2.4	5.0 ± 2.4	6.0 ± 2.8
Mean ± SD	5.6 ± 2.4	5.1 ± 2.4	5.7 ± 2.8
Median	6.0	5.0	6.0
Range	2–9	0–10	2–10
Number of lesion areas			
Mean ± SD	5.1 ± 1.4	5.2 ± 1.2	5.3 ± 1.9
Median	5.0	5.0	5.0
Range	2–9	3–8	0–10

*Abbreviation*: SD, standard deviation

On Day 28 the efficacy (number of dogs free of live mites) was 94.4%, 95.7% and 92.3% for oral fluralaner, topical fluralaner and oral sarolaner, respectively (Table 2). On Days 56 and 84, live mites were not identified in deep skin scrapings on any study dog, therefore the primary efficacy for all groups was 100%.

**Table 2:** Efficacy against *Sarcoptes scabiei* var. *canis* for sarcoptic mange infested dogs treated once with oral or topical fluralaner or with oral sarolaner on Days 0 and 28

	Day 28	Day 56	Day 84
Oral fluralaner (*n* = 54)	94.4%	100%	100%
Topical fluralaner (*n* = 46)	95.7%	100%	100%
Oral sarolaner (n = 26)	92.3%	100%	100%

**Fig. 1:**
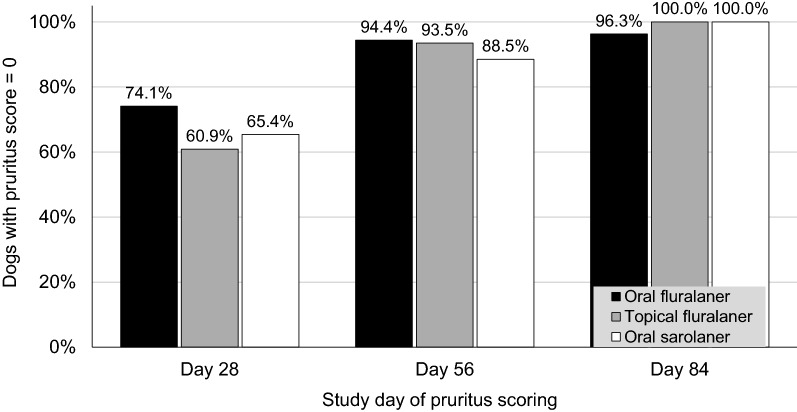
Percentage of dogs with pruritus scores = 0 following one dose of oral or topical fluralaner (Day 0) or two doses of oral sarolaner (Days 0 and 28) for treatment of sarcoptic mange

**Fig. 2:**
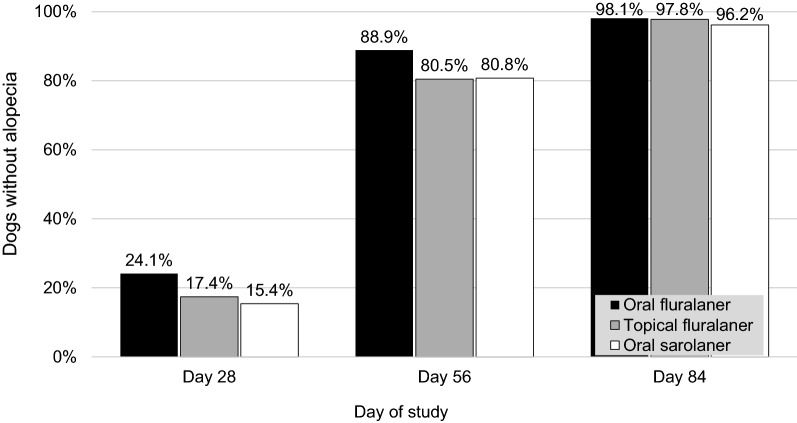
Percentage of dogs without alopecia after one dose of oral or topical fluralaner (Day 0) or two doses of oral sarolaner (Days 0 and 28) for treatment of sarcoptic mange

**Fig. 3:**
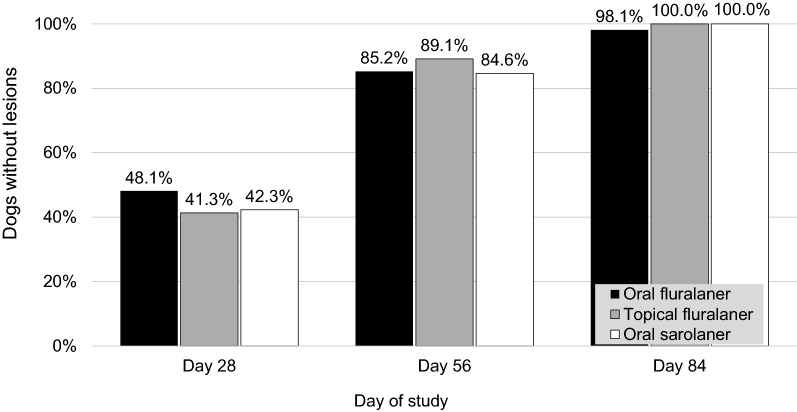
Resolution of skin lesions in dogs after one dose of oral or topical fluralaner (Day 0) or two doses of oral sarolaner (Days 0 and 28) for treatment of sarcoptic mange

A sharp increase in the proportion of dogs with no pruritus (score 0) corresponded with the decline in the number of mite-infested dogs, indicating increasing efficacy (Fig. 1). There was also a marked decline in the severity of alopecia (Fig. 2) and other skin lesions (Fig. 3).

On Day 56, more than 85% of fluralaner-treated dogs and 84.6% of sarolaner-treated dogs were free of lesions (Table 3). In all groups at this visit, there was substantial evidence of hair re-growth with 85% (85 of 100) of fluralaner-treated dogs and 80.8% (21 of 26) of sarolaner-treated dogs having no sign of alopecia.

**Table 3:** Lesion severity (in %) in dogs treated once with oral or topical fluralaner (Day 0) or twice with oral sarolaner (Days 0 and 28). Only the worst lesion was considered in a dog with more than one lesion

	Day 0	Day 28	Day 56	Day 84
Oral fluralaner				
None	0.0	48.1	85.2	98.1
Mild	27.8	48.2	14.8	–
Moderate	48.1	3.7	–	–
Severe	24.1	–	–	1.9^a^
Topical fluralaner				
None	0.0	41.3	89.1	100.0
Mild	13.0	52.2	8.7	–
Moderate	56.5	4.3	2.2	–
Severe	30.5	2.2	–	–
Oral sarolaner				
None	3.9	42.3	84.6	100.0
Mild	7.7	38.5	11.5	–
Moderate	57.7	19.2	3.9	–
Severe	30.7	–	–	–

^a^Skin lesion assessed as trunk pyoderma unrelated to sarcoptic mange

On the last study day (Day 84), dermatological signs of sarcoptic mange had largely resolved in all dogs, and hair coat appeared to have returned to normal in almost all treated dogs as just one dog per group presented with alopecia. At the final assessment, one dog in the oral fluralaner group had a new lesion with alopecia and a pruritus score of 2. Clinical examination of that dog indicated that the signs were due to trunk pyoderma. As the dog had been free of mites at all post-treatment visits, and its pruritus and lesions had completely resolved on Day 56, the new skin lesion was concluded to be unrelated to sarcoptic mite infestation. Median pruritus scores for all groups from Day 28 to Day 84 were zero. At this final visit, all dogs in all groups were considered to be free of lesions caused by sarcoptic mange, and in all groups the pattern of lesion improvement from Day 0 through Day 84 was similar.

## Discussion

The finding of at least one live *S. scabiei* mite in symptomatic dogs on the enrolment day confirmed a positive initial infestation status. By enrolling a much larger number of dogs than the two earlier studies investigating the efficacy of fluralaner against *S. scabiei*, and by continuing observations for 12 weeks following a single treatment, the results confirm the high efficacy of a single dose of oral or topical fluralaner [13, 14]. The efficacy seen following one dose of fluralaner, compared with the need for two treatments with sarolaner, confirms that this convenient approach can be used in treating sarcoptic mange in client-owned dogs.

Control of *S. scabiei* var. *canis* requires treatment of all in-contact dogs [1]. Use of a treatment that requires only a single administration can improve the convenience of mite control in household dogs and reduce the risk that compliance failures could lead to ongoing re-infestation. As *S. scabiei* can survive off the host, the sustained efficacy of fluralaner beyond the mite’s off-host survival period will help to prevent re-infestation of treated dogs from exposure to fomites. A missing or delayed retreatment with a shorter acting treatment, such as oral sarolaner, could lead to a relapse in mite control.

Reliable detection of sarcoptic mites on skin scrapings can make it difficult to definitively demonstrate a complete parasitological cure with this parasite. However, absence of mites on multiple scrapings at the end of the 84-day study, combined with resolution of skin lesions and pruritus supports the conclusion that parasitological cures were achieved.

Live mites were found in all groups on Day 28 indicating that elimination of mites following a single treatment may take longer than 1 month. The most likely explanation for this result is re-infestation from fomites, as in an earlier study mite counts were zero within 28 days following fluralaner treatment [9]. Study conditions in this field trial did not allow assessment of the viability of surviving mites and whether they were recently hatched, feeding or moribund. Regardless of the reason for the identification of mites at 28 days post-treatment, the result confirms that miticidal efficacy against *S. scabiei* should be evaluated beyond one month following treatment, and that efficacy should be confirmed in longer duration field studies.

A study investigating two oral afoxolaner treatments in sarcoptic mange-affected, client-owned dogs in South Africa found efficacy to be 100% at 56 days following the first treatment [18]. At the end of this study, five control dogs treated previously with imidacloprid-moxidectin as a positive control were then treated with afoxolaner. A live mite was found on a skin scraping on one of these dogs 28 days after the afoxolaner treatment. In another field study, afoxolaner was administered on Days 0 and 28, and a live *S. scabiei* mite was seen in a skin scraping from one dog 28 days following each treatment, indicating that parasitological cure was not achieved within 56 days of initial afoxolaner treatment [19]. In a laboratory study in which sarolaner treatments were administered on Days 0 and 28, a live mite was found on one dog 14 days following the second treatment, although this dog was negative on skin scraping 14 days following the initial treatment [20]. In a field study with oral sarolaner, the parasitological cure rate was 88.7% at 30 days following the first treatment and 100% at 30 days following the second treatment [20]. These results indicate that at least two treatments with monthly re-dosed isoxazolines are required to potentially achieve parasitological cure of *S. scabiei* infested dogs.

By Day 28 in the present study, following initial treatment with either isoxazoline, most dogs had a pruritus score of zero. The decline in pruritus scores was concurrent with a marked improvement in the dermatological signs of sarcoptic mange: pruritus, alopecia, crusts/scales, erythema, and papules/pustules. These dermatological improvements were clearly visible at the first post-treatment assessment; however, full recovery in some dogs took longer. Hair regrowth was the longest persisting clinical sign and was not complete in a small proportion of treated dogs at the study conclusion. It is possible that dead mites remaining in the skin continue to induce local irritation or hypersensitivity.

## Conclusions

A single treatment of client-owned, sarcoptic mange-affected dogs with either fluralaner chewable tablets or fluralaner spot-on formulation proved a safe and effective treatment of infestations with *S. scabiei* var. *canis*, maintained through 84 days (12 weeks) after treatment.

## Data Availability

The datasets generated and analyzed during the present study are not publicly available due to confidentially agreements. All original study documentation is archived by the sponsor at MSD facilities (Germany).

## Abbreviation

VICH: Veterinary International Cooperation on Harmonisation
